# Alleviation of migraine related pain and anxiety by inhibiting calcium-stimulating AC1-dependent CGRP in the insula of adult rats

**DOI:** 10.1186/s10194-024-01778-3

**Published:** 2024-05-17

**Authors:** Yang Li, Chenhao Li, Qi-Yu Chen, Shun Hao, Jingrui Mao, Wenwen Zhang, Xun Han, Zhao Dong, Ruozhuo Liu, Wenjing Tang, Min Zhuo, Shengyuan Yu, Yinglu Liu

**Affiliations:** 1 Department of Neurology, The First Medical Center, Chinese PLA General Hospital, Beijing, China; 2 Medical School of Chinese PLA, Beijing, China; 3 School of Basic Medical Sciences, Fujian Medical University, Fuzhou, Fujian Province China; 4 Zhuomin Institute of Brain Research, Qingdao, Shandong Province China; 5 School of Medicine, Nankai University, Tianjin, China; 6 Department of Physiology, Faculty of Medicine, University of Toronto, Toronto, Ontario Canada

**Keywords:** Insular cortex, Chronic migraine, Anxiety behaviors, Adenylate cyclase 1

## Abstract

**Background:**

Recent animal and clinical findings consistently highlight the critical role of calcitonin gene-related peptide (CGRP) in chronic migraine (CM) and related emotional responses. CGRP antibodies and receptor antagonists have been approved for CM treatment. However, the underlying CGRP-related signaling pathways in the pain-related cortex remain poorly understood.

**Methods:**

The SD rats were used to establish the CM model by dural infusions of inflammatory soup. Periorbital mechanical thresholds were assessed using von-Frey filaments, and anxiety-like behaviors were observed via open field and elevated plus maze tests. Expression of c-Fos, CGRP and NMDA GluN2B receptors was detected using immunofluorescence and western blotting analyses. The excitatory synaptic transmission was detected by whole-cell patch-clamp recording. A human-used adenylate cyclase 1 (AC1) inhibitor, hNB001, was applied via insula stereotaxic and intraperitoneal injections in CM rats.

**Results:**

The insular cortex (IC) was activated in the migraine model rats. Glutamate-mediated excitatory transmission and NMDA GluN2B receptors in the IC were potentiated. CGRP levels in the IC significantly increased during nociceptive and anxiety-like activities. Locally applied hNB001 in the IC or intraperitoneally alleviated periorbital mechanical thresholds and anxiety behaviors in migraine rats. Furthermore, CGRP expression in the IC decreased after the hNB001 application.

**Conclusions:**

Our study indicated that AC1-dependent IC plasticity contributes to migraine and AC1 may be a promising target for treating migraine in the future.

**Supplementary Information:**

The online version contains supplementary material available at 10.1186/s10194-024-01778-3.

## Background

Chronic pain exerts an enormous personal and economic burden, affecting more than 30% of people worldwide [1]. Recent reports based on global disease burden analysis show that migraine remains second among the causes of disability, and chronic migraine (CM) affects up to 2% of the global population with limited therapeutic solutions are available [2–4]. According to latest category of chronic pain, the CM is classified into nociplastic pain, the third category that is mechanistically distinct from nociceptive pain, which is caused by ongoing inflammation and damage of tissues, and neuropathic pain, which is caused by nerve damage [5]. The mechanisms that underlie nociplastic pain are not fully understood, but it is thought that augmented central nervous system (CNS) pain and sensory processing and altered pain modulation play prominent roles [5, 6].

The insular cortex (IC) plays an important role as a "hub station" in brain function [7]. It is involved at least in the regulation of emotion, addiction, cognition, taste and vestibular functions [8, 9]. An increasing number of studies have shown that the IC also participates in pain perceptions as the anterior cingulate cortex (ACC) does by influencing synaptic transmission, such as in neuropathic pain, inflammatory pain and phantom limb pain [10–16], which leading these two area into major cortical areas for regulating chronic pain perception, especially for neuropathic pain [17–23]. A brain functional imaging study also has shown that the number of years of CM is significantly correlated with the strength of functional connections between the anterior IC and the medial dorsal thalamus, as well as between the anterior IC and periaqueductal gray matter [24]. Besides, the insular cortical thickness in women with migraine did not thin with age as it does in healthy individuals [25]. Except for the clinical studies, Jia *et al*. found that different frequencies of inflammatory soup (IS)-induced migraine models of rats could cause altered functional connectivity of the IC, which also gave us a clue that the IC may have a potential role in the pathophysiology of migraine [26]. In addition, the IC is an important brain area involved in negative emotion regulation including anxiety and depression [7, 27, 28], which are very common comorbidities among patients with migraine [29, 30].

Our previous studies in naive mice found that bath application of calcitonin gene-related peptide (CGRP), a crucial biomarker in migraine progression, significantly enhanced the excitatory transmission in the ACC and the IC [31, 32], and some studies reported that GluN2B-containing N-methyl-D-aspartate (NMDA) receptors and adenylyl cyclase 1 (AC1) played key roles in chronic pain [16–19]. Liu *et al.* demonstrated that cortical excitatory transmission was significantly enhanced in the ACC of IS-induced migraine model rats, and applying a selective inhibitor of AC1, NB001 in the ACC, could alleviate migraine symptoms [33]. However, less information is available for investigating the synaptic mechanisms about the migraine in the IC under pathological conditions.

Therefore, in the current study, the inflammatory mediators were used to the dura mater of adult rats to establish CM models. We found that the expression of c-Fos and CGRP was increased, and the excitatory transmission as well as phosphorylation of GluN2B receptors in the IC was enhanced. A human-used selective AC1 inhibitor, hNB001, applied locally in the IC or intraperitoneally, showed significant alleviations in the periorbital mechanical threshold and related anxiety behaviors in the rats suffering CM. What’s more, the expression of CGRP in the IC was decreased after applying hNB001. Our study indicated that AC1-dependent IC plasticity contributes to migraine and AC1 may be a promising target for treating migraine in the future.

## Methods

### Animals

Male Sprague-Dawley (SD) rats (8-9 weeks old) were purchased from SiBeiFu Beijing Biotechnology Co., Ltd. (Beijing, China), and housed in a temperature (22-24°C), humidity (40-60%), and light (12 hours light/dark cycle) controlled environment with free access to food and water. The experimental procedures were approved by the Institutional Animal Care and Use Committee, Chinese People’s Liberation Army (PLA) General Hospital, following the Regulations for the Administration of Affairs Concerning Experimental Animals.

### Chronic Migraine (CM) model

Rats were anesthetized with sodium pentobarbital (50 mg/kg) and then fixed in a stereotaxic frame. The scalp covering the dorsal surface was cut to expose the skull. A small hole (1 mm in diameter) was drilled at 1.5 mm to the right of the sagittal suture and 1.5 mm posterior to the bregma for the insertion of a cannula (1 mm in diameter and 1 mm in length; RWD Life Science, China). The dura mater was kept intact during the surgery. The cannula was then fixed with screws and dental cement, and the scalp skin was sutured, leaving only the cannula cap exposed. The rats were then housed individually for postoperative recovery for seven days. The CM model was established by stimulating the dura mater adjacent to the superior sagittal sinus with a “IS”. The IS contained bradykinin (2 mM), histamine (2 mM), serotonin (2 mM), and prostaglandin E2 (0.2 mM), and was dissolved in phosphate buffered saline (PBS) [34]. In awake rats, 10 μL of IS or PBS was infused into the dura mater through the cannula. The infusions were administered over five minutes, once daily, for a total of 14 times.

### Intraperitoneal injection of hNB001

hNB001 is a chemo-synthetic compound (C12H20N6O, Mw=264.33 g/mol) obtained from Forevercheer Holding Ltd.Co. (Hong Kong, China) and is dissolved in normal saline (NS). Upon completion of the CM model, hNB001 (20 mg/kg) or NS was administered via intraperitoneal injection for three consecutive times, once daily.

### Microinjection of hNB001 into the IC

Based on the CM model, cannulas (0.56 mm in diameter; RWD Life Science, China) were also placed in the bilateral IC (5.7 mm left/right, 0 mm anterior to the bregma, and 7.7 mm deep; RWD Life Science, China). The rats were individually housed for one week after the cannula placement. The rest of the operations were the same as the CM model. 1 μL hNB001(10 mg/ml) or NS was slowly injected into the bilateral IC with a microinjector (Hamilton, USA) over five minutes, once a day for three consecutive days. The treatment began on the Day 14, and dural infusion of IS was discontinued.

### Experimental groups

A total of 92 rats were used in the current study. The groups were divided according to the type of drug and the method of administration. Firstly, based on the drug injected into the rat’s dura mater, the groups were divided into the IS group and the PBS group (*n*=20 rats/group, three rats of the IS group were excluded for statistical analysis due to the failure of the surgery or death during the modeling). Secondly, according to the drug received by the IC of the IS rats, the groups were divided into the hNB001 group and the NS group (*n*=14 rats/group). Finally, based on the type of drug injected intraperitoneally into the IS rats, the groups were divided into the i.p. hNB001 group and the i.p. NS group (*n*=12 rats/group).

### Periorbital mechanical threshold test

The periorbital mechanical threshold was measured daily (Day 0-Day 17) using von-Frey filaments (1.0-26 g). Each rat was adapted in the plastic box for 30 minutes before the measurement. During the measurement, the von-Frey filament was applied perpendicularly to the periorbital skin until the filament bent, and was maintained for five seconds or until the rat showed a positive response. The positive response included head withdrawal, vocalization, and pawing at the filament [35]. Three different periorbital locations (lateral canthus, supraorbital and forehead) were stimulated with a filament. Record the weight of the filament when a positive reaction occurs at two or three stimulation points as periorbital mechanical threshold. Repeat this process three times. The filaments were changed from low to high scale. For rats that did not respond to the maximum filament strength (26 g), 26 g was recorded as their periorbital mechanical threshold.

### Hot plate test

Adult rats were placed on the hot plate device (55±0.5°C), surrounded by four plexiglas walls (Cold Hot Plate, Bioseb, USA). The latency to either lick the hind paw or jump was recorded as heat pain thresholds. Before dural infusion, each rat was tested three times with a five-minutes interval between each trial to calculate the average latency, starting one day prior to intervention and repeated every other day. Rats were kept on the hot plate for no more than 20s to avoid thermal injury.

### Behavioral tests

Behavioral studies were performed at set times. Before the experiment, the animals were adapted in the dark behavioral analysis room for at least three hours, with free access to food and water. The animals were placed in the apparatus, and video recording was conducted for five minutes. Within the IS group and the PBS group, rats were tested at the start of the experiment, and on the Day 7 and Day 14. The hNB001 group and NS group were tested before administration (Day 14) and after administration was completed (Day 17), similar to the i.p. hNB001 group and i.p. NS group.

The open field apparatus consisted of a circular black base (120 cm in diameter) surrounded by black walls (40 cm high). The inner chamber was a circle with a diameter of 90 cm, and the outer space was annular. At the beginning of the test, the rats were placed in the central area, facing away from the experimenter. The total distance traveled, the distance covered in the central area, the time spent in the central area, and the number of entries into the central area were recorded by an animal behavior trace analysis system (SuperMaze, Xinruan, China).

The elevated plus maze (EPM) apparatus consisted of two opposite open arms (50 cm x 10 cm) and two opposite closed arms (50 cm x 10 cm x 40 cm). The maze center had a 10 cm x 10 cm open area, and the maze was elevated 60 cm above the ground. At the start of the test, the rats were placed on the central platform of the EPM, facing one of the open arms. The total distance traveled, open arms distance, time spent in the open arms, and the number of entries into the open arms were recorded by the animal behavior trajectory analysis system (SuperMaze, Xinruan, China).

### Immunofluorescence staining and counting

All target tissues were collected 24-28 hours after the last administration. After general anesthesia with sodium pentobarbital, the rats were perfused intracardially with 120 mL of 0.01 M PBS, followed by 120 mL of 4% paraformaldehyde (PFA). The whole brain tissues were fixed in 4% PFA at 4°C for 24 hours, and then dehydrated in 15% and 30% sucrose until the tissues sank to the bottom. The brain tissues were cut into 8 μm thick axial sections on a freezing microtome (Leica, Japan). The sections were permeabilized with 0.25% Triton X-100 mixed with 10% goat serum at room temperature, and then incubated with primary antibodies at 4°C overnight. The sections were washed three times with PBS, each for five minutes, and then incubated with species-specific fluorescently labeled secondary antibodies at 37°C for two hours. Finally, the nuclei were stained with 4’,6-diamidino-2-phenylindole (DAPI, Abcam, ab104139, UK) and mounted. Images were acquired with an upright fluorescence microscope (NIKON ECLIPSE C1, Konni, Japan). The primary antibodies used for immunofluorescence staining were c-Fos (1:2000, cell signaling technology, #2250, USA), CGRP (1:2000, cell signaling technology, #14959, USA), and the secondary antibodies were anti-mouse/rabbit secondary antibodies (1:300, Servicebio, GB21301, GB21303, China). For the detection of positive cells, images were analyzed with ImageJ 1.53t software. Briefly, three regions of the same area were randomly selected in the ACC (0.96-0.72 mm anterior to the bregma), IC (0.24-0.48 mm posterior to the bregma), trigeminal nucleus caudalis (TNC, also known as Sp5c in rats atlas) (14.28-14.40 mm posterior to the bregma), and at 40 times magnification, the number of c-Fos and CGRP positive cells was counted, and then the average value was recorded as the final data.

### Western blotting analysis

Protein preparation and western blotting analysis were performed according to a previous method [36]. Briefly, the bilateral IC brain tissues were collected and stored at -80°C until use. The rat brain tissues were ground at low temperature with a tissue grinder (Servicebio, China), and then fully lysed with RIPA lysis buffer (Beyotime, China) containing protease and phosphatase inhibitors (Beyotime, China). The supernatant was obtained after low-temperature centrifugation. The protein concentration was determined with a BCA kit (AQ, China). Protein (30 μg) was separated by SDS-PAGE gel and electrotransferred to polyvinyl difluoride (PVDF) membranes (Millipor, China). The PVDF membranes were blocked with a quick blocking solution (Yang Guang Bio, China) for one minute, and then incubated with the following antibodies: CGRP (1:1000, cell signaling technology, #14959, USA), GluN2B (1:2000, Proteintech, 21920-1-AP, China), p-GluN2B-S1303 (1:2000, abcam, ab81271, UK), AC1 (1:1000, abcam, ab69597, UK), β-actin (1:3000, Proteintech, HRP-66009, China), and anti-mouse/rabbit secondary antibodies (1:3000, Proteintech, SA00001-1, SA00001-2 China). The protein bands were visualized with the HRP ECL system, and then analyzed with ImageJ 1.53t software for the gray value.

### Brain slices preparation

Coronal brain slices (300 μm) at the level of the IC were prepared using standard methods [31, 33, 37]. Briefly, rats were deeply anesthetized with 5% isoflurane and transcardially perfused with cold cutting solution containing (in mM) 2.5 KCl, 0.5 CaCl_2_, 10 MgSO_4_, 1.25 NaH_2_PO_4_, 2 thiourea, 3 sodium pyruvate, 92 N-methyl-D-glucamine, 20 N-(2-hydroxyethyl) piperazine-N’-2-ethanesulfonic acid (HEPES), 25 D-glucose, 5 L-ascorbic acid, and 30 NaHCO_3_(pH 7.35-7.40). Then the rats were sacrificed by decapitation and the whole brain was removed quickly from the skull and submerged in the oxygenated (95% O_2_ and 5% CO_2_) ice-cold cutting solution. The whole brain tissue was cooled for short time before trimmed as the proper part to glue onto the microslicer (VT1200S Vibratome, Leica, Germany). The coronal brain slices containing the IC were cut, and then incubated in a submerged recovery chamber with artificial cerebrospinal fluid (ACSF) containing (in mM) 124 NaCl, 2.5 KCl, 2 MgSO_4_, 1 NaH_2_PO_4_, 2 CaCl_2_, 25 NaHCO_3_, and 10 D-glucose at room temperature for 1 h. The ACSF was continuously aerated with a mixture of 95% O_2_ and 5% CO_2_.

### Whole-cell patch-clamp recording

Whole-cell recordings were performed in a recording chamber on the stage of an Axioskop 2FS microscope with infrared differential interference contrast optics for visualization. eEPSCs were recorded from layer II/III neurons with HEKA EP10 amplifier (HEKA, Germany), and the stimulations were evoked in layer V of the IC by a bipolar tungsten stimulating electrode. The recording pipettes (3–5MΩ) were filled with the solution containing (in mM) 112 Cs-Gluconate, 5 TEA-Cl, 3.7 NaCl, 0.2 EGTA, 10 HEPES, 2 Mg-ATP, 0.1 Na_3_-GTP and 5 QX-314, which adjusted to pH 7.2 with CsOH and had osmolality of 290 mOsmol. Picrotoxin (PTX,100 μM, Tocris, UK) was presented to block the GABA_A_ receptor-mediated inhibitory synaptic currents. NMDA receptor-mediated EPSCs were recorded at 30 mV by bathing with CNQX (20 mM, Tocris, UK). PEAQX (3 μM, Tocris, UK) was used as GluN2A receptor inhibitor and Ro25-6981 (3 μM, Tocris, UK) was used as GluN2B receptor. Data were discarded if access resistance changed > 15% during an experiment. Data were filtered at 1 kHz, and digitized at 10 kHz.

### Statistical analysis

SPSS 26.0 software (IBM Corp., USA) was used for statistical analysis and Prism 8.3 software was used for mapping. The Shapiro-Wilk’s test was used to analyze data distribution, and Levene’s method was used to check homogeneity of variance. Two-tailed independent sample t-test or two-tailed independent sample rank sum test was used for comparison between two groups. Two-way ANOVA and Repeated measures ANOVA were used with Bonferroni or Least Significant Difference post hoc comparison when the same subjects were measured at three or more time points. When statistical conditions for repeated measures ANOVA were not met, K- independent samples rank sum test was used with Bonferroni post hoc comparison. *P* < 0.05 was considered statistically significant, and data were presented as mean ± standard error of the mean (SEM).

## Results

### Induced CM pain and anxiety behaviors after repeated infusions of IS

**Fig. 1:**
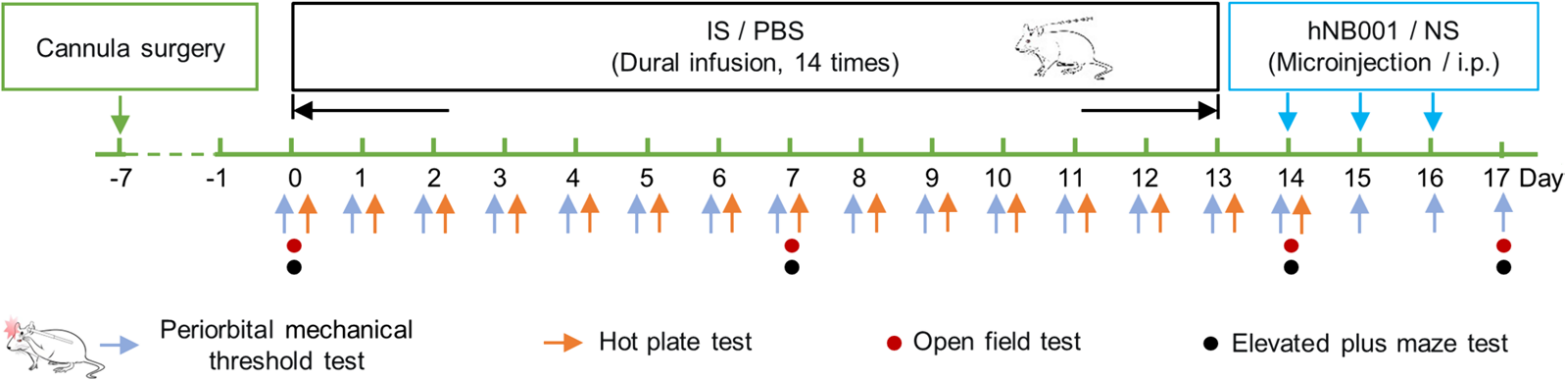
Flow chart of the experimental outline

**Fig. 2:**
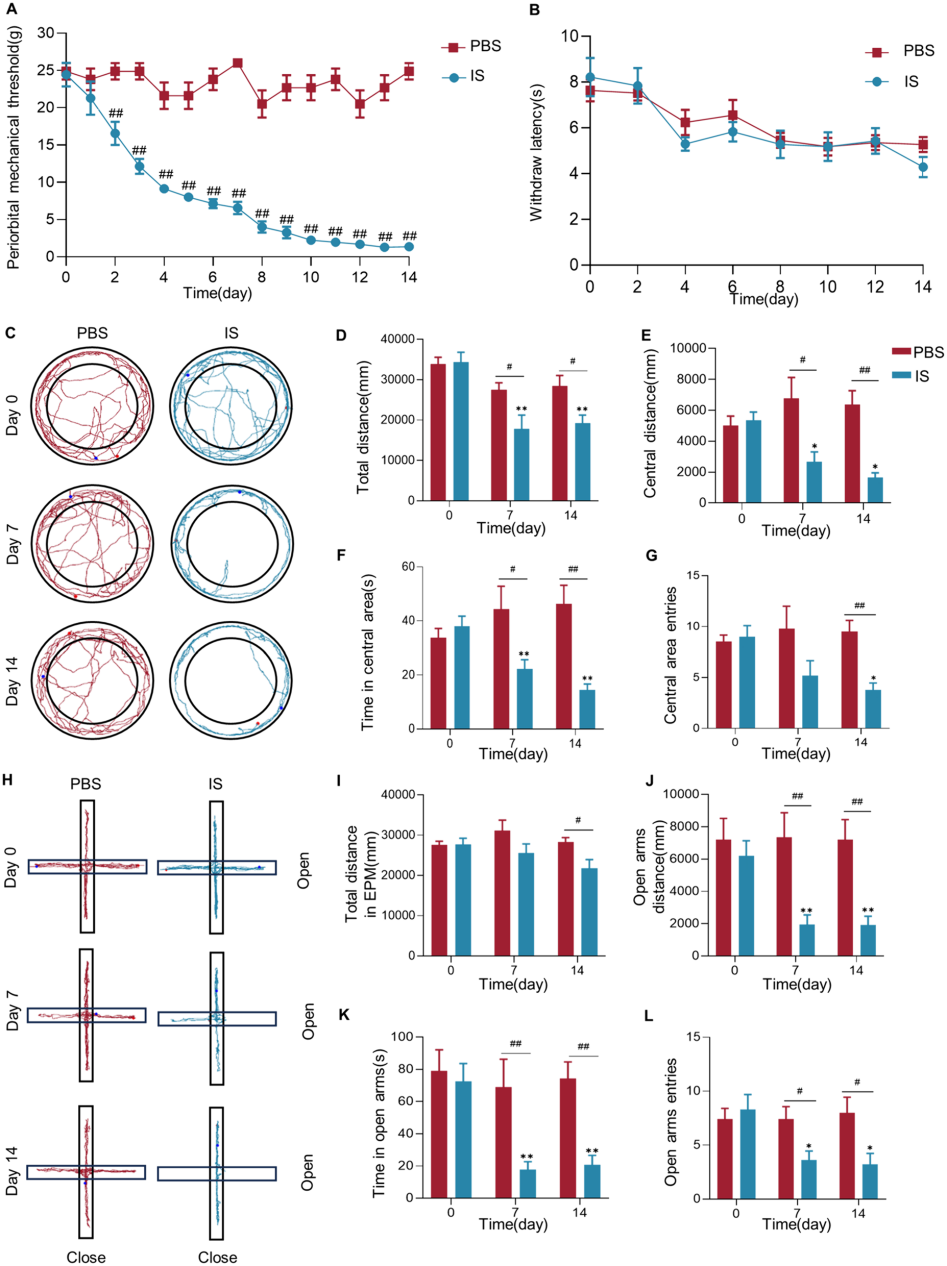
Effects of repeated dural infusions of IS on pain and anxiety behaviors. **A** Decreases in periorbital mechanical threshold in rats were induced by repeated dural infusions of IS (IS: *n*=7 rats, PBS: *n*=10 rats; two-tailed independent sample rank sum test, ##*P*<0.01, compared with the PBS group). **B** Repeated IS injections did not alter thermal pain sensitivity in rat hind paws (*n*=10 rats/group; Day 2, Day 4, Day 8, Day 12 and Day 14, two-tailed independent sample t-test, Day 0, Day 6 and Day 10, two-tailed independent sample rank sum test, compared with the PBS group). **C** Samples of open field test in the IS and PBS rats at different time. **D-G** IS rats showed anxiety behaviors in the open field test (*n*=10 rats/group; two-tailed independent sample t-test, #*P*<0.05, ##*P*<0.01, compared with the PBS group; repeated measures ANOVA with Bonferroni post hoc comparison within IS group, *P<0.05, **P<0.01, compared with the baseline (Day 0)). (**H**) Samples of the EPM test in IS and PBS rats at different time. (**I-L**) IS rats showed anxiety behaviors in the EPM test, but no difference in total distance (*n*=10 rats/group; **I**, **J** (Day 0 and Day 14),** K** and **L** (Day 0), two-tailed independent sample t-test, #*P*<0.05, ##*P*<0.01, compared with the PBS group; **J** (Day 7) and** L** (Day 7 and Day 14), two-tailed independent sample rank sum test, # *P*<0.05, ##*P*<0.01, compared with the PBS group; **I** and** L**, repeated measures ANOVA with Least Significant Difference post hoc comparison within IS group, *P<0.05, **P<0.01, compared with the baseline (Day 0); **J** and** K**, k independent samples rank sum test with Bonferroni post hoc comparison within IS group, *P<0.05, **P<0.01, compared with the baseline (Day 0)). Data are presented as the mean ± SEM

The detailed flow chart of study is shown in Fig. 1. There was no significant difference in the baseline periorbital mechanical threshold between the IS group and the PBS group (*n*(IS)=7 rats, *n*(PBS)=10 rats; Day 0: *P*=0.79; Fig. 2A). However, following two IS injections, the periorbital mechanical threshold in the IS group significantly decreased compared to the PBS group (Day 2: *P*<0.01). This trend continued from Day 3 to Day 14, with the IS group consistently exhibiting a significantly lower threshold than the PBS group ((Day 14: *P*<0.01; Fig. 2A). In the hot plate test, there was no significant difference in hind paw withdrawal latency between the IS and PBS groups (Day 0: *P*>0.99; Day 14: *P*=0.54; Fig. 2B).

At the baseline of the open field test (*n*=10 rats/group), there was no significant difference between the IS group and the PBS group in the total distance (*P*=0.87; Fig. 2C and D), central distance (*P*=0.65; Fig. 2E), time in central area (*P*=0.41; Fig. 2F) and number of central area entries (*P*=0.70; Fig. 2G). On the Day 7, the significant reductions of the total distance (*P*=0.02), central distance (*P*=0.02), and time in the central area (*P*=0.03) were found when compared with the PBS group. On the Day 14, the IS group also showed a significant decrease in the total distance (*P*=0.01), central distance (*P*<0.01), time in the central area (*P*<0.01) and number of central area entries (*P*<0.01) compared with the PBS group (Fig. 2D-G). What’s more, the results within the IS group illustrated significant decrease at Day 7 and Day 14 when compared with baseline in total distance (*P*<0.01), central distance (Day 7: *P*=0.012, Day 14: *P*=0.02), and time in central area (*P*<0.01) (Fig. 2D-F). The number of central area entries only showed significant decreased on the Day 14 compared to the baseline (Day 7: *P*=0.13, Day 14: *P*=0.014; Fig. 2G).

What’s more, at baseline in the EPM test (*n*=10 rats/group), there was no statistical difference between the IS group and the PBS group in the total distance (*P*=0.95; Fig. 2H and I), open arms distance (*P*=0.55; Fig. 2J), open arms time (*P*=0.71; Fig. 2K) and number of entries into the open arms (*P*=0.61; Fig. 2L). On the Day 7, there was no difference between the IS group and the PBS group in the total distance (*P*=0.13; Fig. 2I), but the open arms distance (*P*<0.01), open arms time (*P*<0.01) and number of entries into the open arms (*P*=0.017) were significantly reduced compared to the PBS group (Fig. 2J-L). On the Day 14, the total distance (*P*=0.017), open arms distance (*P*<0.01), open arms time (*P*<0.01) and number of entries into the open arms (*P*=0.015) of the IS group were significantly reduced compared to the PBS group (Fig. 2I-L). Moreover, there were significant reductions in open arms distance (*P*<0.01), open arms time (*P*<0.01) and number of entries into the open arms (*P*<0.01) in the IS group on the Day 7 and Day 14 (Day 7: *P*=0.02, Day 14: *P*=0.04) compared to the baseline (Fig. 2J-L).

### Activation of the IC in a rat model of CM

**Fig. 3:**
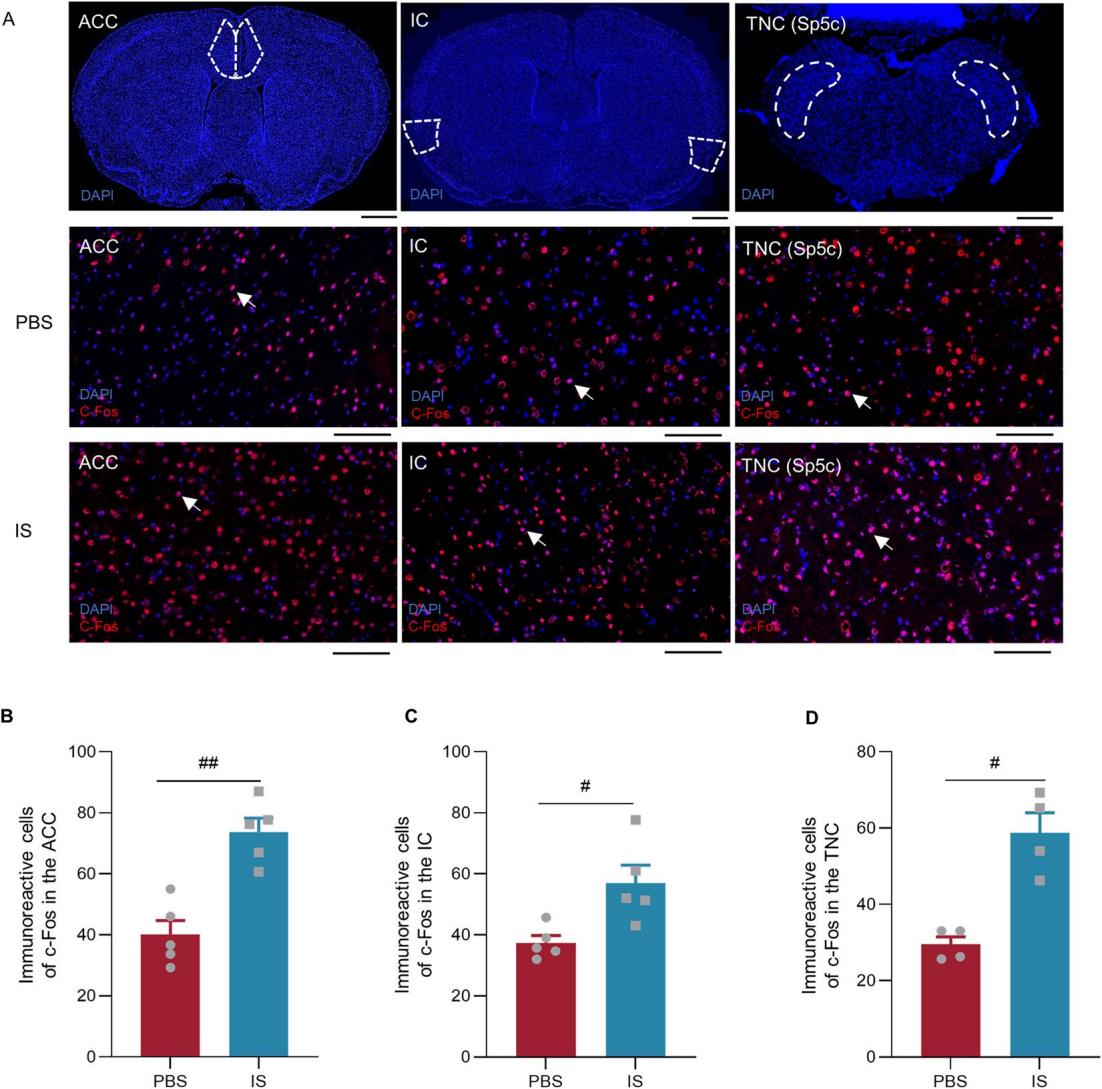
Increased the number of c-Fos positive cells in the ACC, IC, TNC (Sp5c) of IS rats. A Left: Representative coronal section of ACC (top). Bar = 2000 μm. Representative immunofluorescence image for c-Fos in the ACC from PBS group (middle) and IS group (down). Bar = 100 μm. Middle: Representative coronal section of the IC (top). Bar = 2000 μm. Representative immunofluorescence image for c-Fos in the IC from PBS group (middle) and IS group (down). Bar = 100 μm. Right: Representative coronal section of the TNC (Sp5c) (top). Bar = 1000 μm. Representative immunofluorescence image for c-Fos in the TNC (Sp5c) from PBS group (middle) and IS group (down). Bar = 100 μm. The blue color is indicated for DAPI, red color is indicated for c-Fos and positive cells (colocalization) are indicated by white arrows. **B** Increased the number of c-Fos positive cells in the ACC of IS rats (*n*=5 rats/group; two-tailed independent sample t-test). **C** Increased the number of c-Fos positive cells in the IC of IS rats (*n*=5 rats/group; two-tailed independent sample t-test). **D** Increased the number of c-Fos positive cells in the TNC (Sp5c) of IS rats (*n*=4 rats/group; two-tailed independent sample rank sum test). All data are presented as the mean ± SEM (#*P*<0.05, ##*P*<0.01, IS vs. PBS)

To identify whether the IC was activated in the migraine process, the immunofluorescence staining was used to detect the expression of c-Fos protein among the trigeminovascular system. We found that the positive cells of c-Fos protein in the ACC (*n*=5 rats/group; *P*<0.01), IC (*n*=5 rats/group; *P*=0.015) and TNC (*n*=4 rats/group; *P*=0.02) were significantly increased in the IS group compared with the PBS group (Fig. 3A and B).

### Long-term potentiation of excitatory synaptic transmission in the IC

**Fig. 4:**
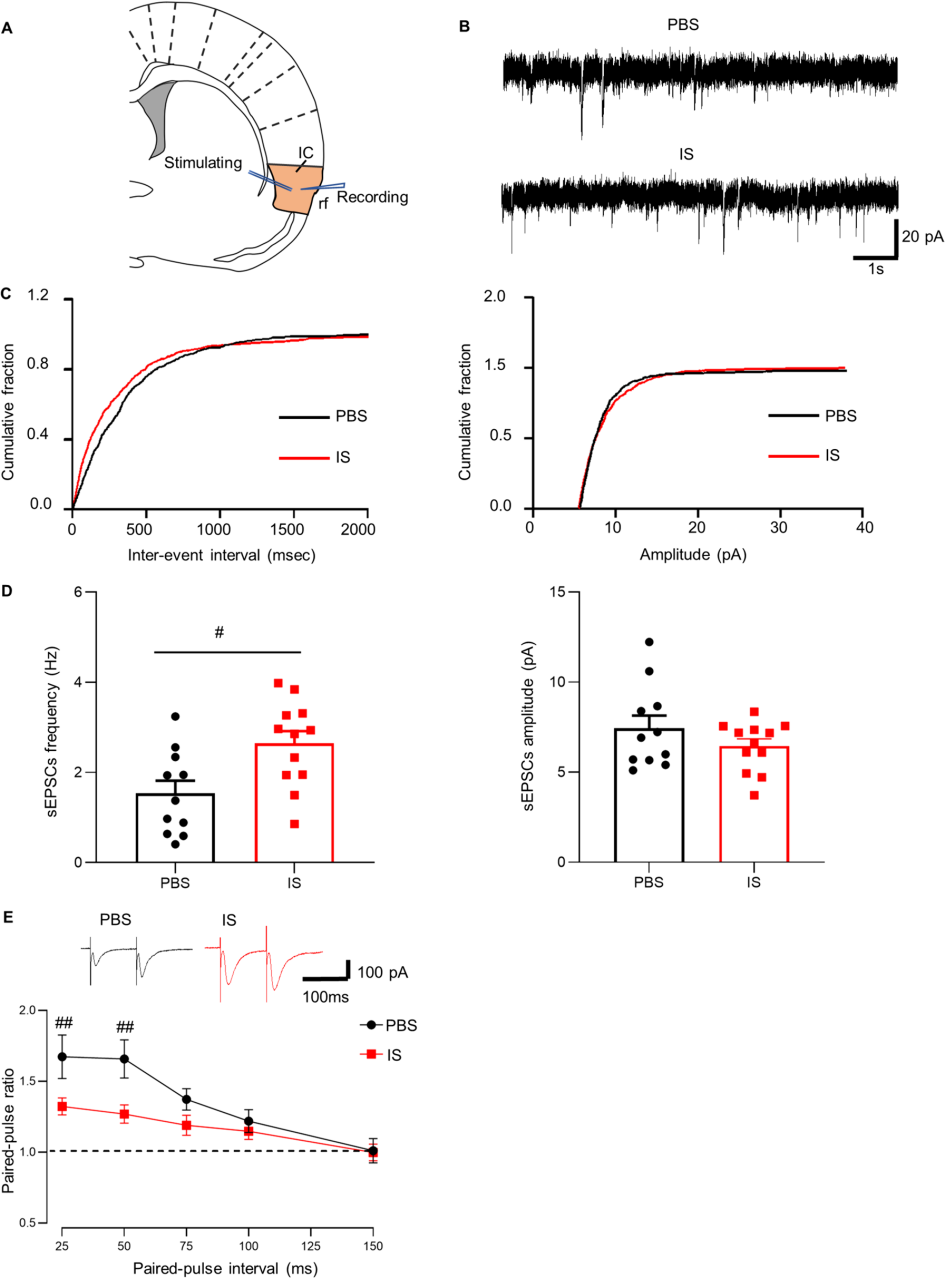
Increased synaptic transmission in the IC area of IS rats. **A** Schematic diagram showed the placement of stimulating and recording electrodes in the IC. rf, rhinal fissure. **B** Representative traces of the sEPSCs recorded in the IC neurons from IS and PBS rats. **C** Cumulative fraction of inter-event interval (left) and amplitude (right) of the sEPSCs in PBS rats and IS rats. **D** Statistic results of the frequency (IS: *n*=12 neurons/5 rats, PBS: *n*=11 neurons/6 rats; two-tailed independent sample t-test; left) and amplitude (IS: *n*=12 neurons/5 rats, PBS: *n*=11 neurons/6 rats; two-tailed independent sample t-test; right) of sEPSCs. (**E**) Representative traces with an interval of 50 ms recorded in the IC (top). Paired-pulse ratio (the ratio of EPSC2/EPSC1) was recorded at intervals of 25, 50, 75, 100, 125 and 150 ms from PBS and IS rats (IS: *n*=11 neurons/6 rats, PBS: *n*=11 neurons/5 rats; two-way ANOVA with Bonferroni post hoc; down). All data are presented as the mean ± SEM (##*P*<0.01, IS vs. PBS)

The schematic diagram and representative recording diagram for spontaneous excitatory postsynaptic currents (sEPSCs) in the IC were shown in Fig. 4A- C. The frequency of sEPSCs was significantly increased in IS rats compared with PBS rats (*n*(IS)=12 neurons/5 rats, *n*(PBS)=11 neurons/6 rats; *P*=0.01; Fig. 4D left). While, the amplitude of sEPSCs did not show significant differences between two groups (*n*(IS)=12 neurons/5 rats, *n*(PBS)=11 neurons/7 rats; *P*=0.22; Fig. 4D right). Moreover, paired-pulse facilitation (PPF) is a transient form of plasticity commonly used as a measure of presynaptic function, in which the response to the second stimulus is enhanced as a result of residual calcium in the presynaptic terminal after the first stimulus [38]. In IS rats, PPF was observed at different stimulus intervals of 25, 50, 75, 100, 125 and 150 ms. At the stimulus intervals of 25 and 50 ms, there were significant reductions in PPF in the IC neurons compared with those from PBS rats (*n*(IS)=11 neurons/6 rats, *n*(PBS)=11 neurons/5 rats; 25ms intervals: *P*<0.01; 50ms intervals: *P*<0.01; Fig. 4E).

**Fig. 5:**
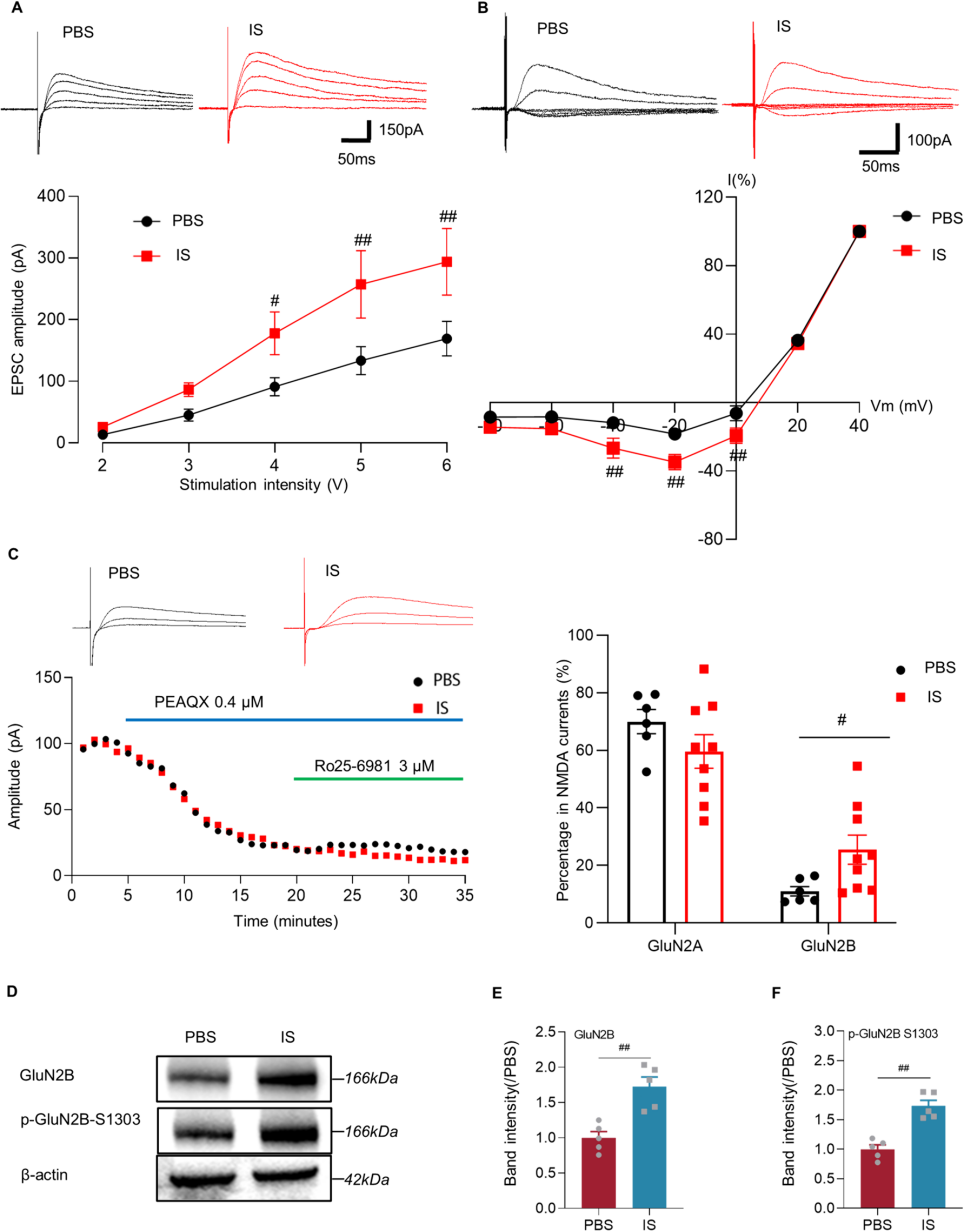
Enhanced NMDA receptors-mediated currents and their expression in the IC. **A** The synaptic input-output curve in slices from IS rats was steeper than in those from PBS group (IS: *n*=10 neurons/6 rats, PBS: *n*=11 neurons/5 rats; two-way ANOVA with Bonferroni post hoc). **B** I-V curves of NMDAR-EPSCs of IC pyramidal neurons recorded at holding potentials ranging from − 80 to + 40 mV in PBS and IS rats (IS: *n*=12 neurons/5 rats, PBS: *n*=10 neurons/5 rats; two-way ANOVA with Bonferroni post hoc). **C** A timeline plot of one representative neuron in IC showing the contribution of NMDA GluN2A and GluN2B -mediated currents (left). PEAQX (0.4 μM): selective GluN2A antagonist, Ro25-6981 (3 μM): selective GluN2B antagonist. Summarized data showing the contribution of GluN2A (two-tailed independent sample t-test) and GluN2B (two-tailed independent sample rank sum test) -mediated currents in IC neurons (IS: *n*=9 neurons/6 rats, PBS: *n*=6 neurons/5 rats; right). **D** Representative western blotting for GluN2B and p-GluN2B-S1303 in the IC from IS and PBS rats. **E** The total protein levels of GluN2B, p-GluN2B-S1303 significantly enhanced in IS rats (*n*=5 rats/group; two-tailed independent sample t-test). **F** The phosphorylation level of GluN2B, p-GluN2B-S1303, significantly enhanced in IS rats (*n*=5 rats/group; two-tailed independent sample t-test). All data are presented as the mean ± SEM (#*P*<0.05, ##*P*<0.01, IS vs. PBS)

To determine the receptor associated with the enhanced transmission in the IC of IS rats, we recorded the input (stimulation intensity)-output (EPSC amplitude) efficiency and I-V relationship of the NMDA receptor-mediated synaptic responses. The synaptic input-output curve in slices from IS rats was steeper than in those from the PBS group (*n*(IS)=10 neurons/6 rats, *n*(PBS)=11 neurons/5 rats; 4v intensity: *P*=0.04; 5v intensity: *P*<0.01; 6v intensity: *P*<0.01; Fig. 5A). Meanwhile, I-V curves of NMDA receptor-mediated EPSCs of the IC pyramidal neurons recorded at holding potentials ranging from − 80 to + 40 mV in PBS and IS rats (*n*(IS)=12 neurons/5 rats, *n*(PBS)=10 neurons/5 rats; -40mv: *P*<0.01; -20mv: *P*<0.01; 0mv: *P*<0.01; Fig. 5B). These results suggested that NMDA receptor-mediated synaptic transmission is enhanced in IS rats. Furthermore, we used PEAQX, a specific GluN2A subunit containing NMDA receptor antagonist, and Ro 25-6981, an antagonist of GluN2B, to investigate whether the ratio of the two receptor subtypes changed in the IC neurons between the IS and PBS groups. After recording the total NMDA receptor-mediated EPSCs in the IC neurons, GluN2A and GluN2B subunit-mediated currents were isolated by sequential application of the GluN2A-specific antagonist PEAQX and the GluN2B-specific antagonist Ro 25–6981 (Fig. 5C left). Within both IS group and PBS group, there was no difference in the GluN2A percentage in NMDA receptors-mediated currents in the IC neurons, while, the GluN2B percentage was higher in IS group than that in the PBS group (*n*(IS)=9 neurons/6 rats, *n*(PBS)=6 neurons/5 rats; GluN2A percentage: *P*=0.21; GluN2B percentage: *P*=0.018; Fig. 5C right). To further identify the above changes of the IC area after IS-modelling, the expression of GluN2B receptor in the IC area was detected. The expression of GluN2B, as well as its phosphorylation was higher in the IS group than that in the PBS group (*n*=5 rats/group; GluN2B: *P*<0.01; p-GluN2B-S1303: *P*<0.01; Fig. 5D-F).

### AC1 is required for increases in CGRP and anxiety behaviors

**Fig. 6:**
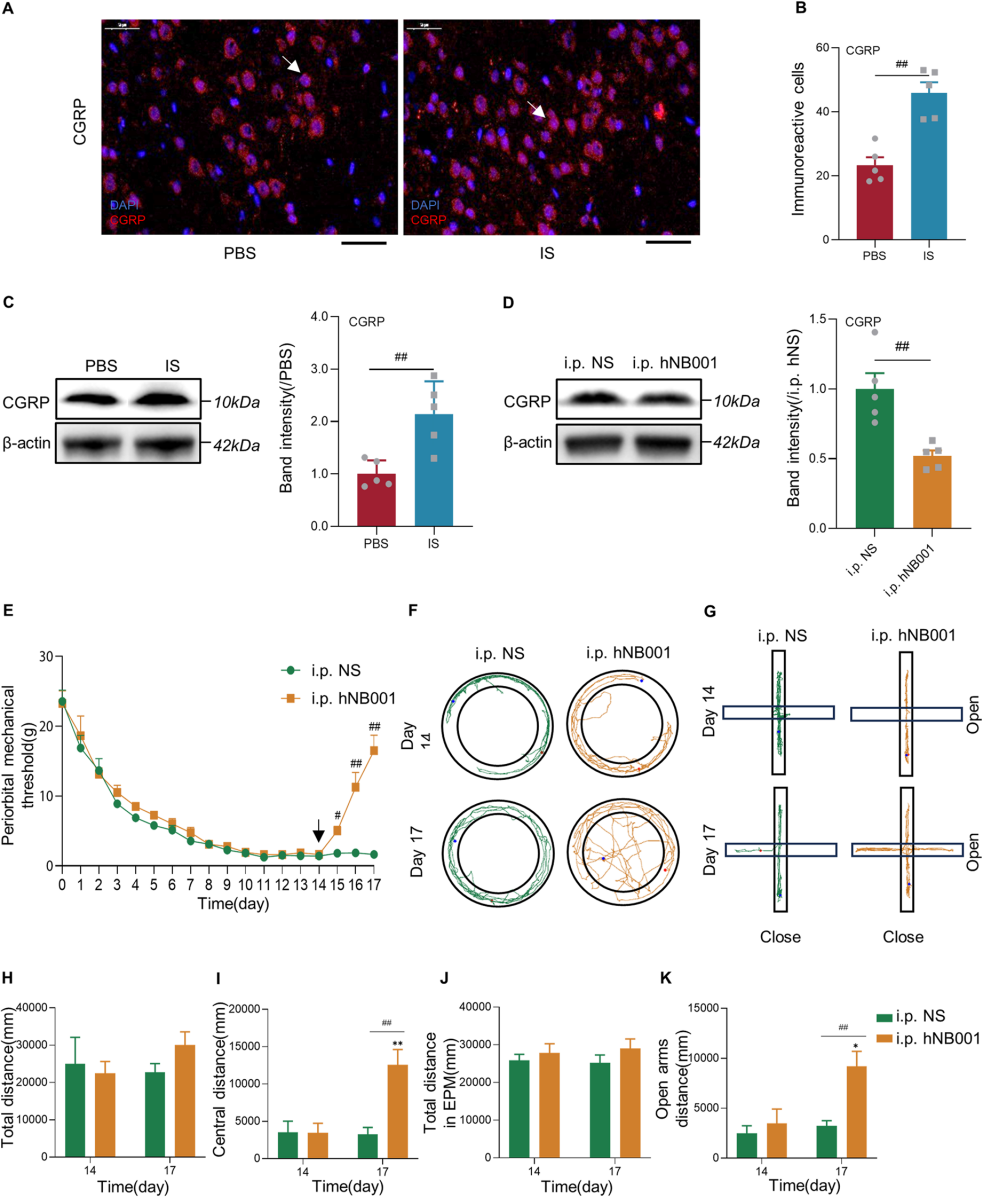
AC1 is required for increases in CGRP and anxiety behaviors. **A** Representative immunofluorescence image for CGRP in the IC from PBS group (left) and IS group (right). Bar = 50 μm. The blue color is indicated for DAPI, red color is indicated for CGRP and positive cells (colocalization) are indicated by white arrows. **B** Increased the number of CGRP positive cells in the IC of IS rats (*n*=5 rats/group; two-tailed independent sample t-test). **C** Representative western blotting band for CGRP in the IC from IS and PBS groups(left). The total protein levels of CGRP significantly enhanced in the IC of IS group (*n*=5 rats/group; two-tailed independent sample t-test; right). **D** Representative western blotting band for CGRP in the IC from i.p. NS and i.p. hNB001 groups (left). The total protein levels of CGRP significantly reduced in the IC of i.p. hNB001 group (*n*=5 rats/group; two-tailed independent sample t-test; right). **E** The hNB001 reversed the decrease in periorbital mechanical threshold in the IS rats (*n*=8 rats/group; two-tailed independent sample rank sum test). The black arrow indicates the first intraperitoneal injection of hNB001. **F** Samples of open field test from IS rats that received NS or hNB001 injections. **G** Samples of EPM test from IS rats that received NS or hNB001 injections. **H, I** In the open field test, hNB001 increased the distance traveled in the central area of IS rats, but had no effect on the total distance traveled (*n*=6 rats/group; two-tailed independent sample t-test). **J, K** In the EPM test, hNB001 increased the distance traveled in the open arms of IS rats, but had no effect on the total distance traveled (*n*=6 rats/group; two-tailed independent sample t-test). All data are presented as the mean ± SEM (#*P*<0.05, ##*P*<0.01, IS vs. PBS or i.p. hNB001 vs. i.p. NS; *P<0.05, **P<0.01, compared with Day 14)

Both immunofluorescence staining and western blotting tests showed that the expression of CGRP in the IC area increased among rats suffering CM (immunofluorescence staining: *n*=5 rats/group, *P*<0.01, Fig. 6A, B; western blotting tests: *n*=5 rats/group, *P*<0.01, Fig. 6C). The human-used AC1 inhibitor, hNB001, was applied intraperitoneally into the IS group, and the following western blotting results showed that the expression of CGRP in the IC of i.p. hNB001 group were significantly decreased compared with the control (*n*=5 rats/group; *P*<0.01; Fig. 6D). Besides, the periorbital mechanical threshold in the i.p. hNB001 group was significantly higher than that in the i.p. NS group after once injection (*n*=8 rats/group; Day 15: *P*=0.014; Fig. 6E). From Day 16 to Day 17, the threshold of the i.p. hNB001 group continued to increase and was significantly higher than that of the i.p. NS group (Day 16: *P*<0.01; Day 17: *P*<0.01; Fig. 6E). Furthermore, the effects of intraperitoneal injection of hNB001 on anxiety behaviors were detected by the open field test and the EPM test (Fig. 6F and G). In the open field test on Day 17, although there was no difference in total distance (*n*=6 rats/group; *P*=0.11; Fig. 6H), the central distance of the i.p. hNB001 group was significantly more than that of the i.p. NS group (*P*<0.01; Fig. 6I). Within the i.p. hNB001 group, the central distance on Day 17 was significantly more than that on Day 14 (*P*<0.01; Fig. 6I). For EPM test, there was no difference in total distance (*n*=6 rats/group; *P*=0.25; Fig. 6J), but the open arms distance (*P*<0.01; Fig. 6K) of the i.p. hNB001 group was significantly longer than that of the i.p. NS group on Day 17. After three times of intraperitoneal injection of hNB001, the above parameters were significantly increased compared with Day 14 (*P*=0.016; Fig. 6K).

### Inhibition of AC1 in the IC alleviated migraine pain and anxiety behaviors

**Fig. 7:**
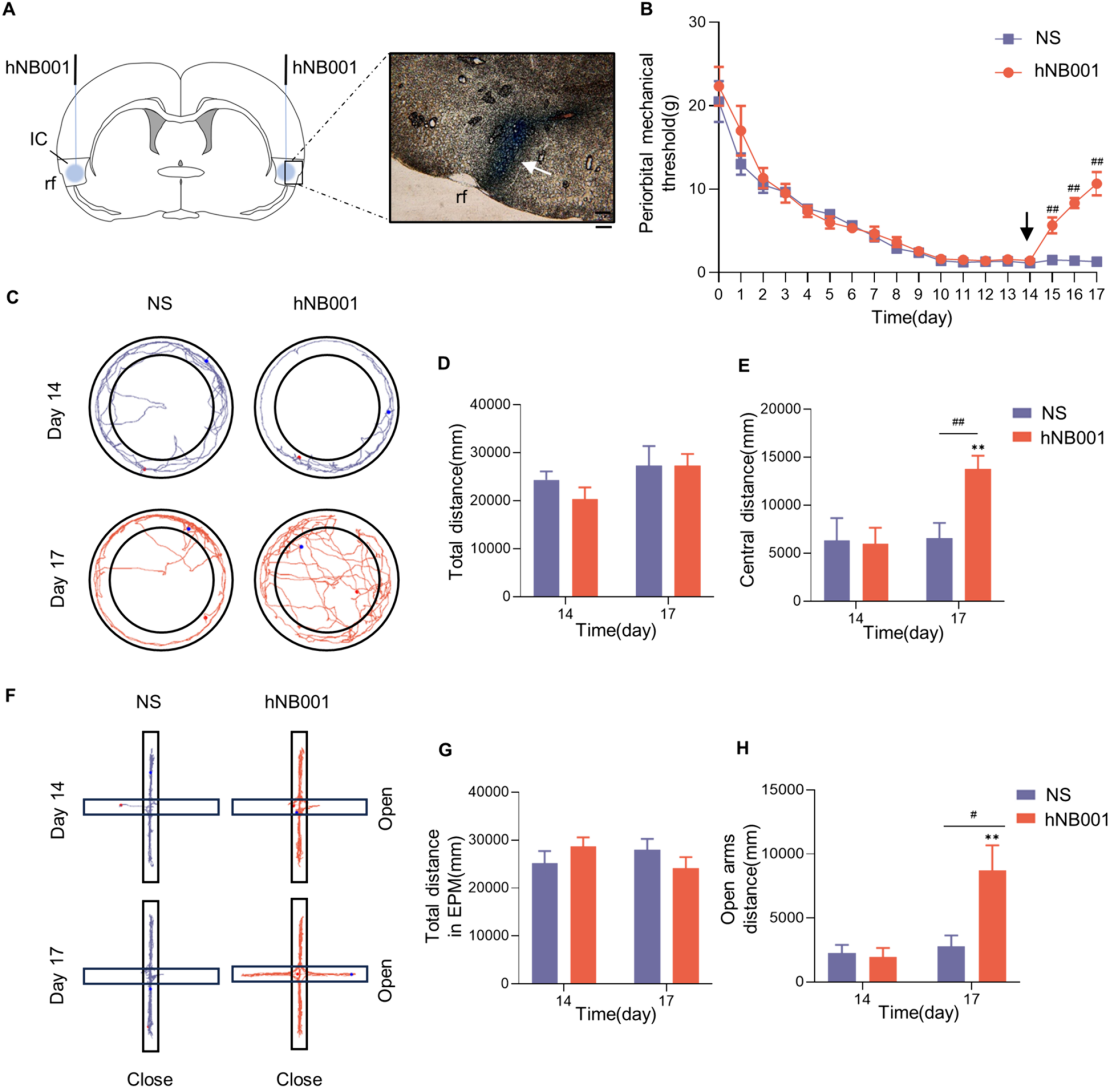
Inhibition of AC1 in the IC alleviated migraine pain and anxiety behaviors. **A** Schematic diagram showed the placement of hNB001 microinjection in the IC (left), and microscopy images of coronal rat brain tissue section after prussian blue staining showing the placement (white arrow, right). Bar=100 μm. **B** The hNB001 reversed the decrease in periorbital mechanical threshold in the IS rats (*n*=6 rats/group; two-tailed independent sample rank sum test). The black arrow indicates the first hNB001 administration. **C** Samples of the open field test from IS rats that received NS or hNB001 microinjections. **D, E** In the open field test, hNB001 increased the distance traveled in the central area of IS rats, but had no effect on the total distance traveled (*n*=6 rats/group; two-tailed independent sample t-test). **F** Samples of EPM test from IS rats that received NS or hNB001 microinjections. **G, H** In the EPM test, hNB001 increased the distance traveled in the open arms of IS rats, but had no effect on the total distance traveled (*n*=6 rats/group; two-tailed independent sample t-test). All data are presented as the mean ± SEM (**P<0.01, compared with Day 14; #*P*<0.05, ##*P*<0.01, hNB001 vs. NS)

After microinjecting hNB001 into the bilateral IC area (Fig. 7A), the periorbital mechanical threshold of the hNB001 group significantly increased starting from the first injection (*n*=6 rats/group; Day 15: *P*<0.01; Fig. 7B). Next, anxiety-like behaviors were detected. The open field test showed no differences on total distance (*n*=6 rats/group; *P*=0.99; Fig. 7C, D), but the central distance of the hNB001 group was significantly increased compared with NS injections (*P*<0.01; Fig. 7C-E). The EPM results indicated that after hNB001 microinjection in the IC area, there was no difference in total distance (*n*=6 rats/group; *P*=0.26; Fig. 7F, G), but the open arms distance increased when compared with the NS group (*P*=0.02; Fig. 7F-H).

## Discussion

Here, we find that dural repeated IS stimulations in rats induce migraine-like symptoms and related anxiety behaviors. The integrated data of biochemical, physiological and behavioral tests demonstrate that the IC area is activated under the pathological conditions of chronic migraine. The excitatory transmission in the IC enhanced, and the NMDA receptor mediated responses, especially the GluN2B-contaning NMDA receptor mediated responses increased. Furthermore, a selective AC1 inhibitor, hNB001, attenuated the increased expression of CGRP in the IC, and applied locally or intraperitoneally of hNB001 alleviate pain threshold as well as anxiety behaviors in rats suffering CM.

### Activation of insula after IS-induced migraine condition

Based on Moskowitz and Ashina’s proposals, the initiation of migraine depends on activation and sensitization of first-order trigeminovascular neurons and the afferent fibers of these neurons innervate the meninges and its vessels [2, 39]. Thus, according to previous researches, continuous dural infusion of IS were used to induce meningeal nociception and activate the trigeminal neurovascular pathway, resulting in the orofacial allodynia [35, 40]. A significant decrease in the periorbital mechanical threshold is observed in rats after continuous IS stimulations. Compared with our previous studies, we find that the IS injections for 14 days are sufficient to induce stable pain status in adult rats [40, 41]. At the same time, the expression of c-Fos, a reliable marker of neuronal activation after stimulation [42] , and CGRP, a crucial biomarker in migraine progression [43], are increased in the IC area compared with control group, which demonstrated the IC is involved in processing migraine modeling in the rats. Concurrently, through the open field test and the EPM test, we observe a significant increase in anxiety-like negative emotions in IS rats on Day 7 and Day 14. Since the periorbital mechanical threshold is decreased from twice injections of IS, it indicates the co-occurrence of migraine and its related anxiety behaviors. These results are consistent with clinical observations that migraine patients often suffer from anxiety and/or depression [44, 45].

### Insular excitability and chronic migraine

The cumulative studies from human and animal studies suggest that migraine is a disease of brain dysfunction, including increased cortical excitability, structural and/or functional changes in the brainstem as well as multiple brain regions [46]. Previous studies indicate that glutamate significantly influences the regulation of pain and negative emotions [17, 47, 48]. It has been shown that glutamate levels rise in the cerebrospinal fluid of migraine patients during and between attacks [49–51]. In our study, we find that the excitatory transmission in the IC of migraine rats enhanced, and the NMDA receptor mediated responses, especially the GluN2B-contaning NMDA receptor mediated responses increased. Some studies have shown that the phosphorylation of NMDA GluN2B receptors contributes to cortical excitation and chronic pain of neuropathic and visceral pain [16, 52]. In this study, we reveal the expression and phosphorylation of GluN2B-contaning NMDA receptor are upregulated in the IC of rats suffering CM, which are coincident with the research about the ACC area in CM models [33]. These results indicate that both IC and ACC area may share the similar signaling pathways in regulating migraine processing.

### The role of AC1-involved signaling pathway of insula in the chronic migraine

Previous studies have demonstrated that AC1 is primarily expressed in neurons, and no AC1 gene expression was found in heart, liver, or kidney cells [53, 54]. Morphological studies have proved that in the forebrain, there is no detectable differences of the expression of AC1 in the ACC, IC, somatosensory cortex, thalamus, spinal dorsal horn, and dorsal root ganglia [55, 56]. AC1 has been shown in our previous studies to be involved in the regulation of synaptic excitability as a critical downstream regulatory molecule of the NMDA receptor pathway [16, 53, 57]. Here, the intraperitoneal injection of hNB001 is used as an approach of peripheral application. It shows that hNB001 could alleviate orofacial allodynia as well as anxiety-like behavior by decreasing the expression of CGRP in the IC. Besides, hNB001 is a small molecule substance. Both of these indicate that hNB001 could cross the blood -brain barrier. While, we cannot rule out the possibility that peripheral application of hNB001 could also affect brain regions (ACC, amygdala, etc.) that expressing AC1 and modulating pain as well as its related emotions. Thus, the microinjection of hNB001 into the IC is further applied to show the direct effect of the IC area in migraine progression. The data shows similar analgesic and anxiolytic effects, which further support that the hNB001 could pass the blood-brain barrier and exert its effect in the brain. These also have been reported in a few previous studies[58, 59]. The elevated expression of CGRP in the IC is attenuated after intraperitoneally and locally treating with hNB001 (Fig. 6 and Fig. S3), which indicates that regulation of AC1 could influence the expression of CGRP, leading to the pain management. These demonstrate that AC1-dependent IC plasticity contributes to chronic migraine.

### Clinical implications

Nowadays, CGRP receptor antagonists, anti-CGRP antibodies and anti-CGRP receptor antibodies have proved effective for relieving migraine symptoms, strongly supporting that CGRP has a major role in migraine pathophysiology [2, 60–62] .However, use of monoclonal antibodies against CGRP or its receptor still has potential immunogenicity and theoretical concerns of cardiovascular safety and liver toxicity, which limited the clinical application [61]. Some CGRP or CGRP receptors monoclonal antibodies are difficult to penetrate the blood-brain barrier and need to be injected in time. This method of administration creates inconvenience and risks during the injection process [61, 63]. However, the hNB001 we used in the current study can be absorbed orally and has exhibited notable analgesic effects in other animal models, such as cancer pain, neuropathic pain and phantom limb pain, with little effect on physiological pain perception, cognition, and motor function. Particularly, it does not show obvious addiction [64–66]. Besides, the results of a clinical phase I study have been published, confirming the safety and good tolerance of hNB001 in human [67]. Consequently, the hNB001 has a potential prospect for application in treating migraine in the future.

### Limitations

Although our study provides strong evidence that the IC is involved in processing of migraine and related anxiety behaviors, there are still some limitations needed further exploration. For instance, the only male rats were used in the current study in order to exclude the effect of estrogen fluctuations. Previous studies found that the majority of changes in the expression of CGRP and its receptor were present in both the IS and PBS-control treated male and female rats and the changes might be much more significant within female groups than in male groups [68]. Considering the present results have already shown that male rats have significant differences among groups, the female rats are not added in view of animal welfare and ethics. Secondly, the upstream and downstream molecular signals of AC1 are still unclear and the functional connections between the IC and other brain regions also require further investigations. Lastly, the methods and timing of hNB001 administrations could also be further explored to identify an optimal mode.

## Conclusions

In summary, our findings reveal that the GluN2B receptor and its downstream molecule AC1 in the IC area play crucial roles in chronic migraine, and inhibition of AC1 can effectively alleviate pain and related anxiety behaviors. AC1 may become a potential target for the treatment of migraine in the future.

## Fundings

All studies were funded by the National Natural Science Foundation of China (82101288, YLL) and the National Natural Science Foundation of China (81901134, HX).

### Supplementary Information

**Additional file 1:** **Figure S1. **The raw western blotting images for the GluN2B and p-GluN2B-S1303 in the IC from the IS and PBS rats (*n*=5 rats/group). 

**Additional file 2: Figure S2.  **The raw western blotting images for CGRP among different groups. (**A**) The raw western blotting images for CGRP in the IC from the IS and PBS rats (*n*=5 rats/group). (**B**) The raw western blotting images for CGRP in the IC from the i.p. hNB001 and i.p. NS rats (*n*=5 rats/group). (**C**) The raw western blotting images for CGRP in the IC from the hNB001 and NS rats (*n*=5 rats/group).

**Additional file 3: Figure S3. **Microinjection of the hNB001 in the IC decreased the expression of CGRP in the IC. (**A**) Representative western blotting band for CGRP in the IC from the NS and hNB001 groups. (**B**) The total protein levels of CGRP significantly reduced in the IC of the hNB001 group (*n*=5 rats/group; two-tailed independent sample t-test; *P*<0.01). All data are presented as the mean ± SEM (##*P*<0.01, hNB001 vs. NS).

## Data Availability

No datasets were generated or analysed during the current study.

## Abbreviations

AC1: adenylate cyclase 1
ACC: anterior cingulate cortex
ACSF: artificial cerebrospinal fluid
CGRP: calcitonin gene-related peptide
CM: chronic migraine
CNS: central nervous system
DAPI: 4’,6-diamidino-2-phenylindole
EPM: elevated plus maze
EPSCs: excitatory postsynaptic currents
IC: insular cortex
IS: inflammatory soup
NMDA: N-methyl-D-aspartate
NS: normal saline
PBS: phosphate buffered saline
PFA: paraformaldehyde
PLA: People’s Liberation Army
PPF: paired-pulse facilitation
PTX: picrotoxin
PVDF: polyvinyl difluoride
SEM: standard error of the mean
sEPSCs: spontaneous excitatory postsynaptic currents
TNC: trigeminal nucleus caudalis
