# Supplementary figures and images for: Alleviation of migraine related pain and anxiety by inhibiting calcium-stimulating AC1-dependent CGRP in the insula of adult rats
Source: J Headache Pain. 2024 May 17;25(1):81. doi: 10.1186/s10194-024-01778-3 (PMC11100092; doi:10.1186/s10194-024-01778-3)

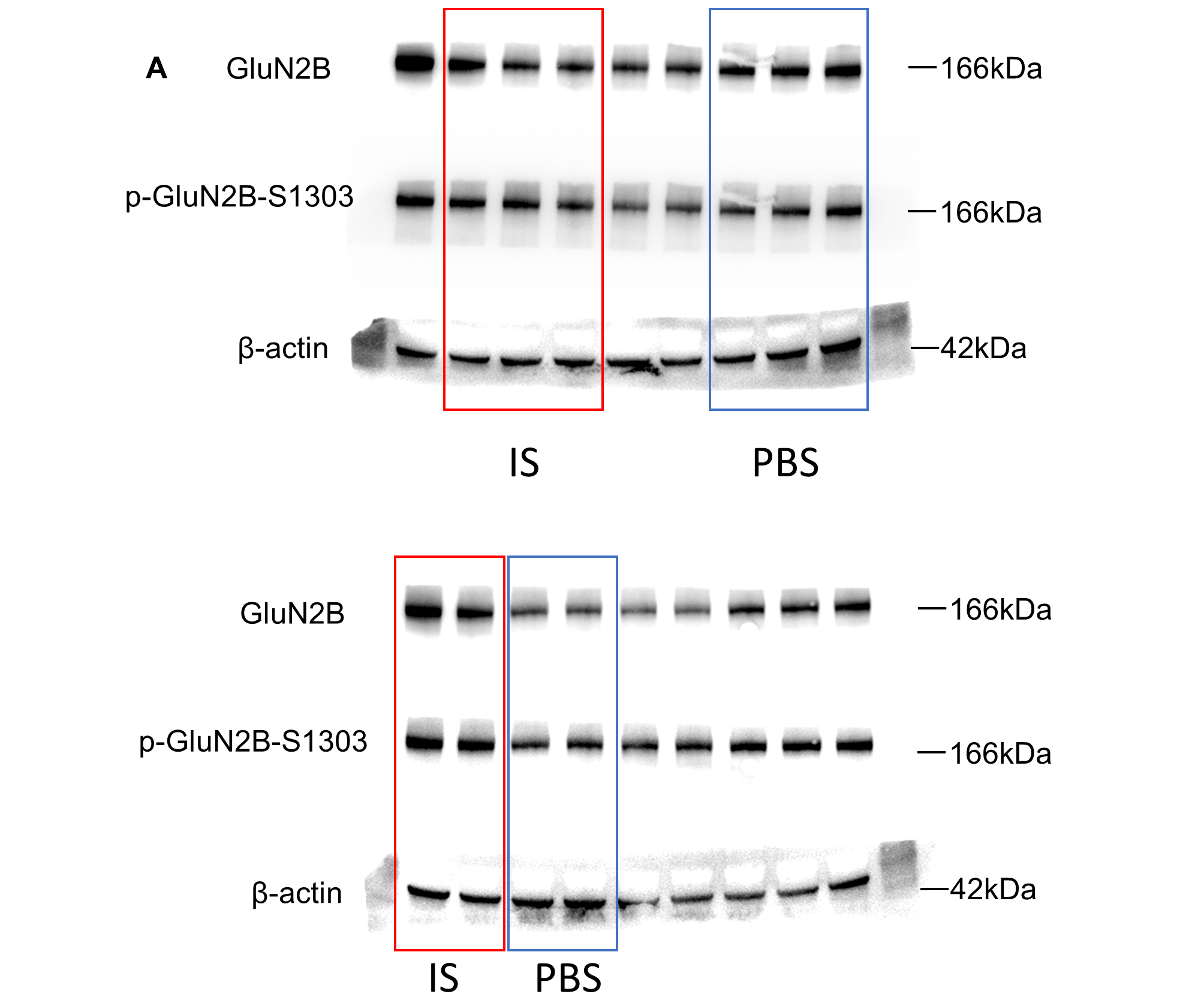

Supplement: Supplementary file 1 — Additional file 1: Figure S1. The raw western blotting images for the GluN2B and p-GluN2B-S1303 in the IC from the IS and PBS rats (n=5 rats/group). [file 10194_2024_1778_MOESM1_ESM.tif]

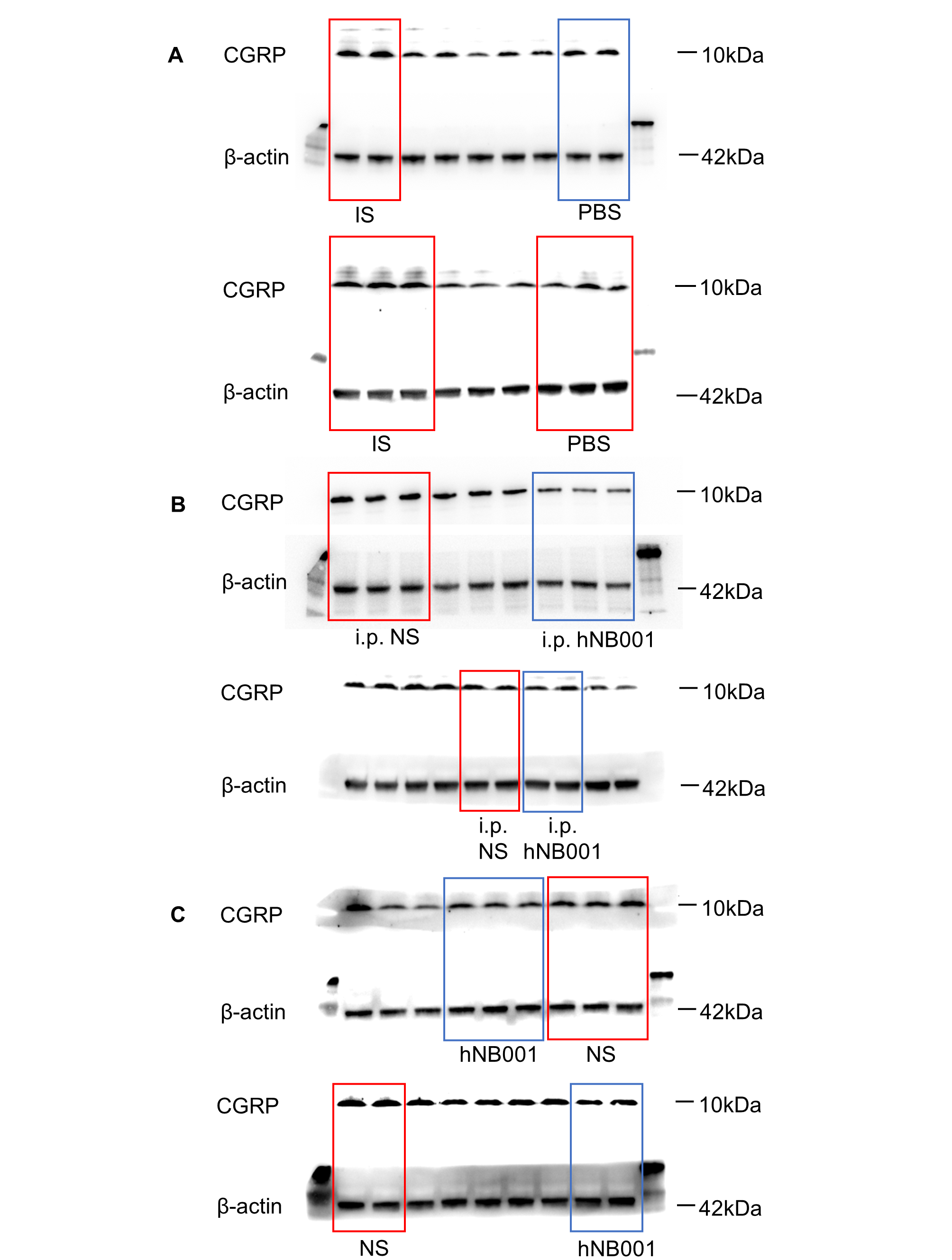

Supplement: Supplementary file 2 — Additional file 2: Figure S2. The raw western blotting images for CGRP among different groups. (A) The raw western blotting images for CGRP in the IC from the IS and PBS rats (n=5 rats/group). (B) The raw western blotting images for CGRP in the IC from the i.p. hNB001 and i.p. NS rats (n=5 rats/group). (C) The raw western blotting images for CGRP in the IC from the hNB001 and NS rats (n=5 rats/group). [file 10194_2024_1778_MOESM2_ESM.tif]

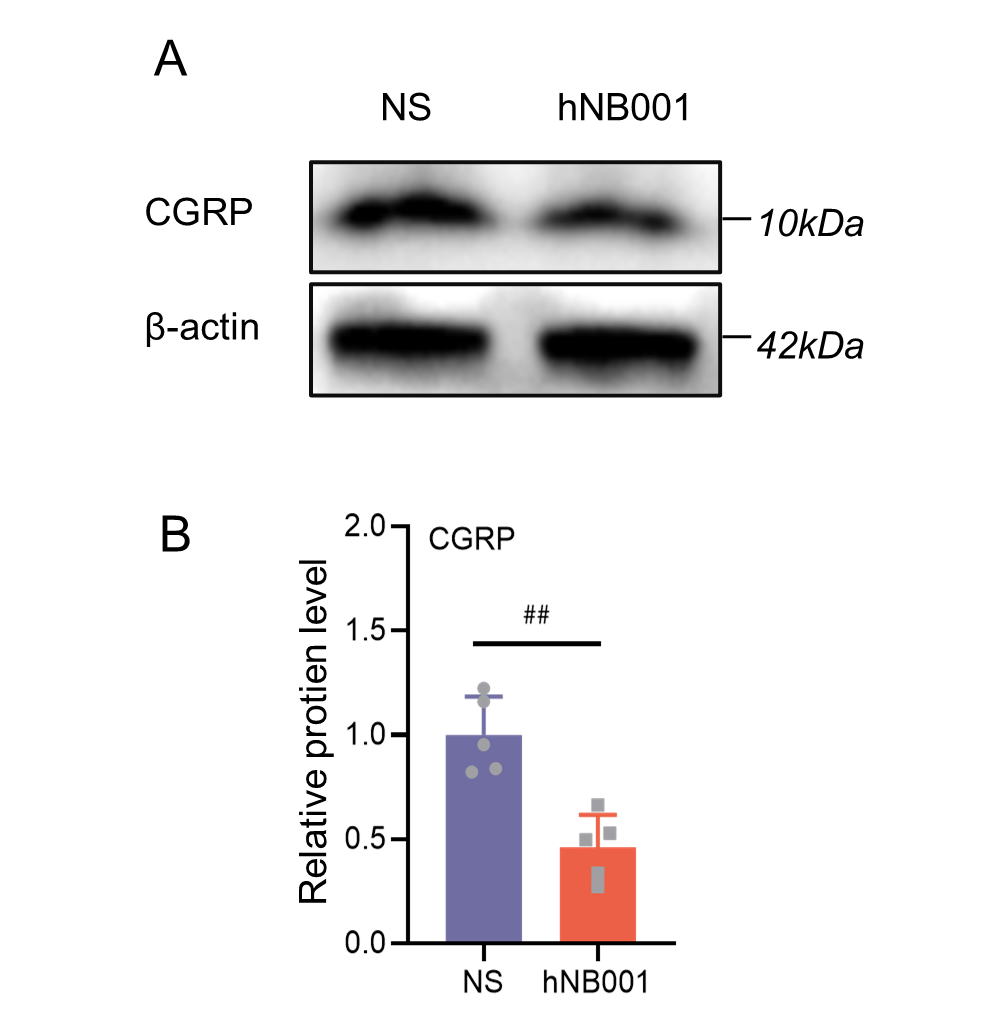

Supplement: Supplementary file 3 — Additional file 3: Figure S3. Microinjection of the hNB001 in the IC decreased the expression of CGRP in the IC. (A) Representative western blotting band for CGRP in the IC from the NS and hNB001 groups. (B) The total protein levels of CGRP significantly reduced in the IC of the hNB001 group (n=5 rats/group; two-tailed independent sample t-test; P<0.01). All data are presented as the mean ± SEM (##P<0.01, hNB001 vs. NS). [file 10194_2024_1778_MOESM3_ESM.tif]
